# The Adverse Effects of Methoxsalen on The Oogenesis of Balb/C Mice

**Published:** 2013-11-20

**Authors:** Mona Farhadi, Esmaeil Fattahi, Homa Mohseni Kouchesfahani, Abbas Shockravi, Kazem Parivar

**Affiliations:** 1 Department of Biology, Faculty of Agriculture, Islamic Azad University, Saveh Branch, Saveh, Iran; 2 Department of Biology, Islamic Azad University, Ayatolah Amoli Branch, Amol, Iran; 3 Department of Zoology, School of Biological Sciences, Kharazmi University, Tehran, Iran; 4 Department of Chemistry, Faculty of Chemistry, Kharazmi University, Tehran, Iran

**Keywords:** Methoxsalen, Ovaries, Estrogen, Abnormality, Follicles

## Abstract

**Objective:**

Methoxsalen is a natural photoactive compound which is found in many seed plants. A number of epidermal proliferative disorders can be treated by methoxsalen along with long wave ultraviolet A (UVA).

**Materials and Methods:**

In an experimental study, we aimed to demonstrate the effect of methoxsalen, UVA and their combination on oogenesis Balb/C mice. There were two experimental groups and a control group. The experimental groups were composed of i. a short term group with treatment duration of 15 days and ii. a long term group with treatment duration of 5 weeks. Both the long term and short term experimental groups were further subdivided into a UVA group, a methoxsalen group and a methoxsalen plus UVA group. After treatment, mature females in prosterus phase of ovarian cycle were scarified with ether, while their ovaries were removed and prepared for histological studies.

**Results:**

Both macro and microscopic studies showed significant anomalies (p<0.05) among experimental group ovaries as compared to control group. The obtained results showed a significant decrease in the following factors: number and diameter of corpus lutei, Graafian follicles, diameter of granulosa cell layer and oocytes, number of primordial،and primary and growing follicles, while we observed an increase in number of atretic follicle. Furthermore, our findings confirmed an increase in theca diameter only through UVA treatment. Methoxsalen also reduced circulating estrogen levels in blood serum, significantly. Other cases of teratogenecity, such as follicles with three oocytes and disorganization in corpus luteum cells were observed.

**Conclusion:**

The result suggests that UVA, methoxsalen and their combination cause health problems and cell injuries.

## Introduction

The psoralens are metabolite substances which are found in fruits and vegetables. The methoxsalen is a photoactive compound, so it can be considered as psoralens or family group. The chemical name of methoxsalen is 9-methoexy-7H- (3.2 g) furo benzopyran- 7-one. Methoxsalen is widely used in treatment of a number of epidermal proliferative disorders (such as leprosy, vitiligo and psoriasis) (1-6). The psoralens have been reported as mutagenic and carcinogenic agents associated with a number of adverse effects (3-19). Methoxsalen causes skin cancer, chromatid exchange, chromosomal aberrations, gene mutation in humans (9) and apoptotic effect on some blood cells (4)."The combination treatment regimen of psoralen and ultra violet radiation of 320-400 nm wavelength commonly referred to as ultraviolet A (UVA) is known by the acronym, psoralen + UVA treatment (PUVA)".

The drug reaches its maximum bioavailability 1.5-3 hours after injection and may be last up to 8 hours (6-8). Methoxsalen is known to have biochemical reaction with DNA. Photo activation causes methoxsalen to conjugate and to make covalent bonds with DNA. This, in turn, triggers production of mono functional and bi functional adducts. In mono functional adducts, a single strand of DNA is added, whereas bi functional adducts is defined as a cross liking of psoralen to both strands of DNA (10). Studies on the mechanism of the interaction between 8-methoxy-psoralen (MOP) and DNA have showed that at low doses, 8-MOP follows an endothermic process and combines with DNA as an intercalator, while at higher drug loads, it takes an exothermic process in order to bind to the outside of DNA, somewhere in the minor groove and compacting the DNA (8-10). Methoxsalen, containing a reversible bound to serum albumin, is preferentially absorbed by epidermal cells. The psoralens have been reported to modulate plasma melatonin levels in rats and human. Methoxsalen is able to induce apoptosis in different cell and tissue types including lung adenocarcinoma (3), and B lymphocytes (8). Backstrom and Wetterbry (11) has showed that mehoxsalen increases melanin and N-acetylserotonin of pineal gland in cell culture of rat, significantly psoralen and UVA light (PUVA) *in vivo* therapy has more effect on the spermatocytes than oocyte or embryo while methoxsalen alone causes severe toxicity (13). PUVA therapy induces skin tumors, carcinoma, papiloma, epiderm cancer, fibrosarcomas, and squamous cell carcinoma on mouse (12-15). Studies on developing mouse embryo in days 7,8 and 9 of gestation have showed methoxsalen and UVA cause teratogenic effects on embryo, including embryo mortality, small embryo, exencephaly, skeletal malformation, a decrease in body weight and crown-rump (CR) of embryo, as well as a decrease in weight and diameter of placenta (16). Diawara et al. (12,13) demonstrated that taking an overdose of xanthotoxin and bergapten significantly reduced the number of implantation sites, pups, and corpora lutea in the experimental animals in comparison with the control group. Moreover, there was a significant reduction in full and empty uterine weight. Depending on the dose of these compounds, xanthotoxin and bergapten were also reported to reduce circulating estrogen levels, significantly.

## Materials and Methods

In an experimental study, female Balb/C mice (28 days old) were obtained from the animal house of Department of Biology at Kharazmi University. Methoxsalen were obtained from Aldrich Chemical Company, USA, while different derivatives of this family were synthesized by new methods at the Organic Chemistry Laboratory of Tarbiate Moallem University. Methoxsalen solvent was Tween 80 solution (Sigma-P4780) while LD50 value was determined by 160 mg/kg body weight. Solution was injected intraperitonealy in a long period of time (three times a week during five weeks), and after two hours, the mice were exposed to UVA radiation of 0.034 j/cm2. Both the long term and short term experimental groups were further subdivided into a UVA group, a methoxsalen group and a methoxsalen plus UVA group. After 2 hours of exposition to 0.034 j/cm2 UVA, methoxsalen plus UVA group received methoxsalen (80 mg/kg body weight).

Mature females (60 days old) in proestrus phase (highest blood estrogen levels) of ovarian cycle were sacrificed with ether and the ovaries of both experimental and control groups were removed and prepared for histological studies. For each animal, information about body weight and relative ovaries weight were recorded at the beginning of experiment and at the end of week 5 (on day of sacrificed).

The number and diameter of corpora lutea, the number and diameter of Graafian follicles, diameter of ovary, granulosa cell layer, oocyte-granulosa- theca, number of primordial, primary and growing follicles, atretic follicles, as well as circulating blood estrogen levels were also measured in each series of experiments.

### Statistical analysis

All data were analyzed using SPSS version 15 and general linear model of analaysis of variance (ANOVA). For each variable, mean was calculated of confidence intervals at the 5% level using Duncan’s new multiple range tests.

## Results

Macroscopic studies demonstrated number and diameter of Graafian follicles reduced in all treatments (methoxsalen, UVA and methoxsalen plus UVA) as compared with control group in long and short term injection, significantly (Figes1,2); however, an increase in diameter of Graafian follicle was only observed in UVA treatment in short term injection (Fig 2A, B).

**Fig 1:**
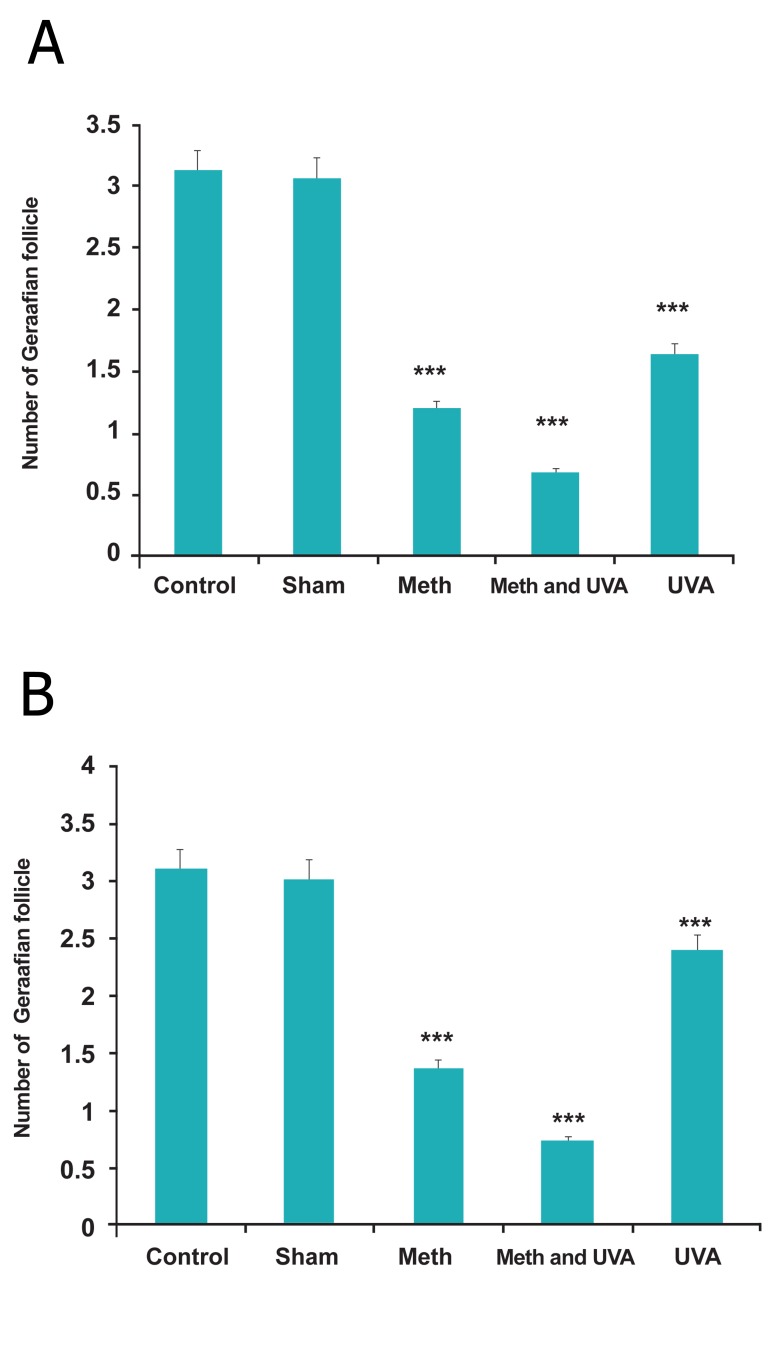
A. Comparision number of Graafian follicle in long term methoxsalen injection with and without UVA (***p<0.001). B. Comparision number of Graafian follicle in short term methoxsalen injection with and without UVA (***p<0.001).

Also, thickness of granolusa layer, number and thickness of corpus luteum reduced in all treatment groups, significantly (Figes3,4,5) and also changes in the number of primordial and atretic follicels in the presence and absence of UVA in long and short term treatment was significant (Figes6,7).

**Fig 2:**
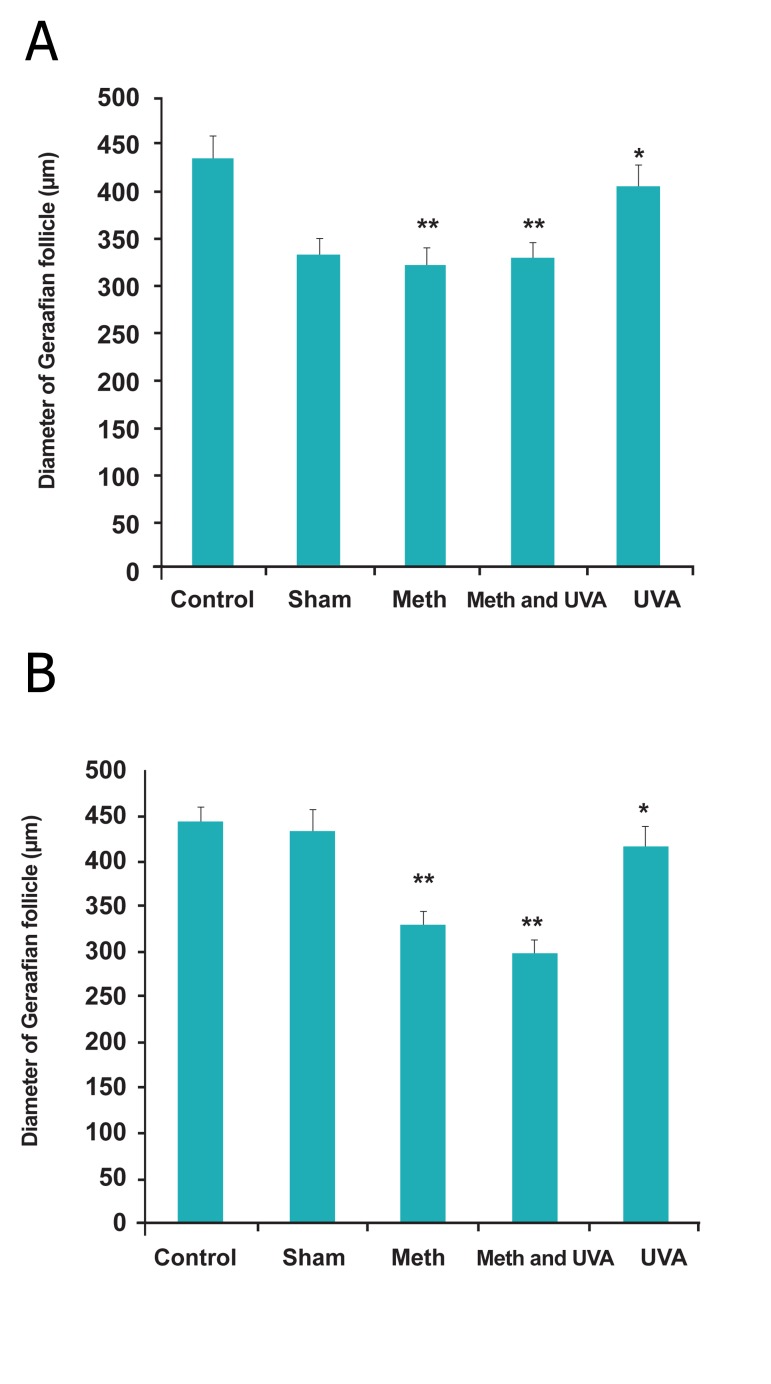
Comparision diameter of Graafian follicle in long term methoxsalen injection with and without UVA (*p<0.05, **p<0.01). B. Comparision diameter of Graafian follicle in short term methoxsalen injection with and without UVA (*p<0.05, **p<0.01).

**Fig 3:**
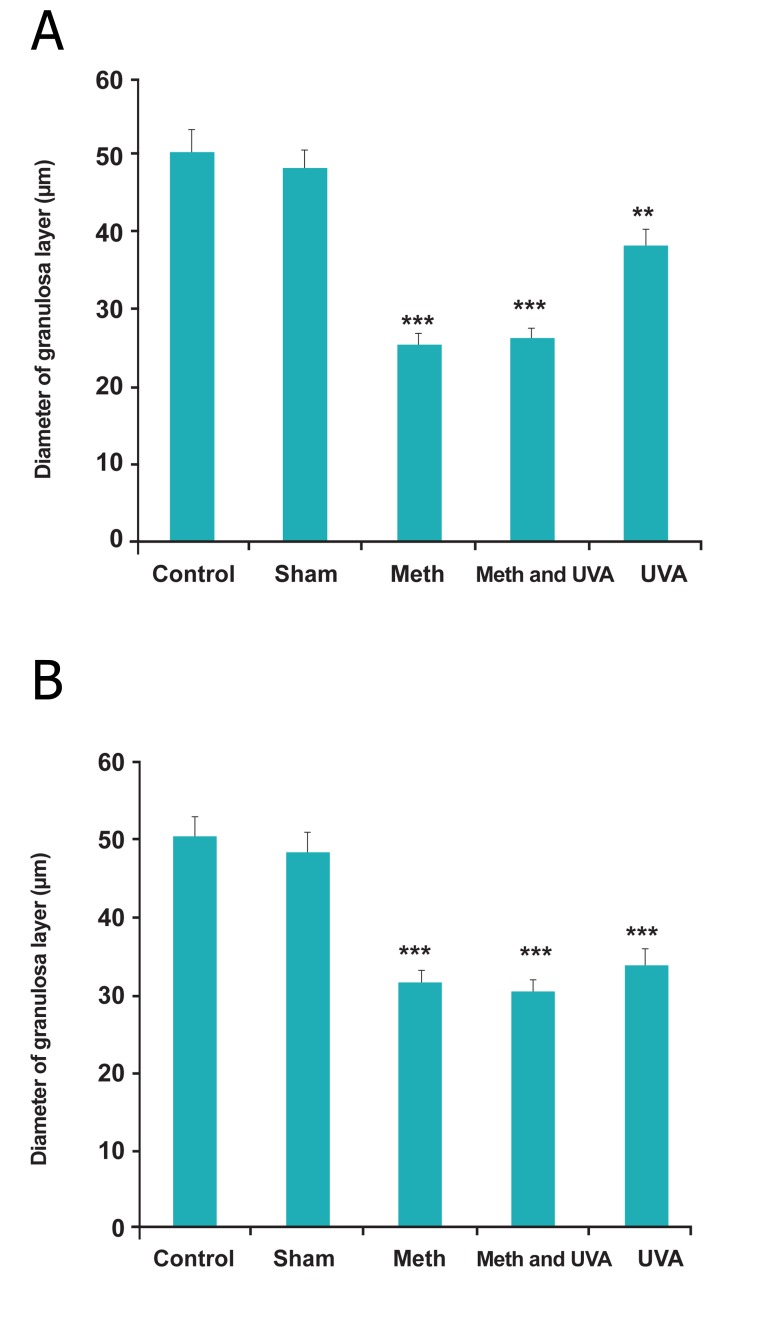
A. Comparision thickness of granulosa layer in long term methoxsalen injection with and without UVA (*p<0.01, p<00.1). B. Comparsion thickness of granulosa layer in short term methoxsalen injection with and without UVA (***p<0.001).

**Fig 4:**
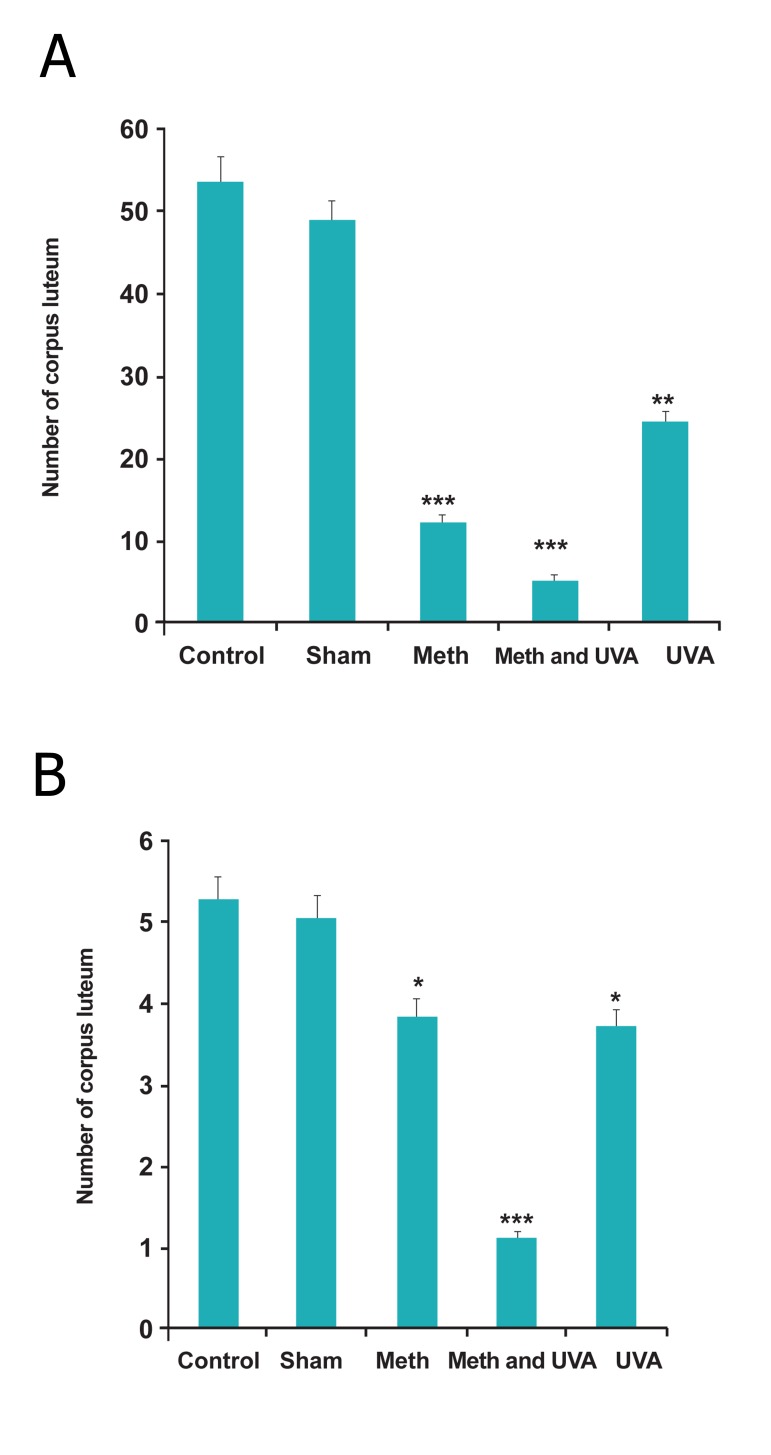
A. Comparision number of cotpus luteum in Long term methoxsalen injection with and without UVA (**p<0.01, ***p<0.001). B. Comparision number of cotpus luteum in short term methoxsalen injection with and without UVA (*p<0.05, ***p<0.001).

**Fig 5:**
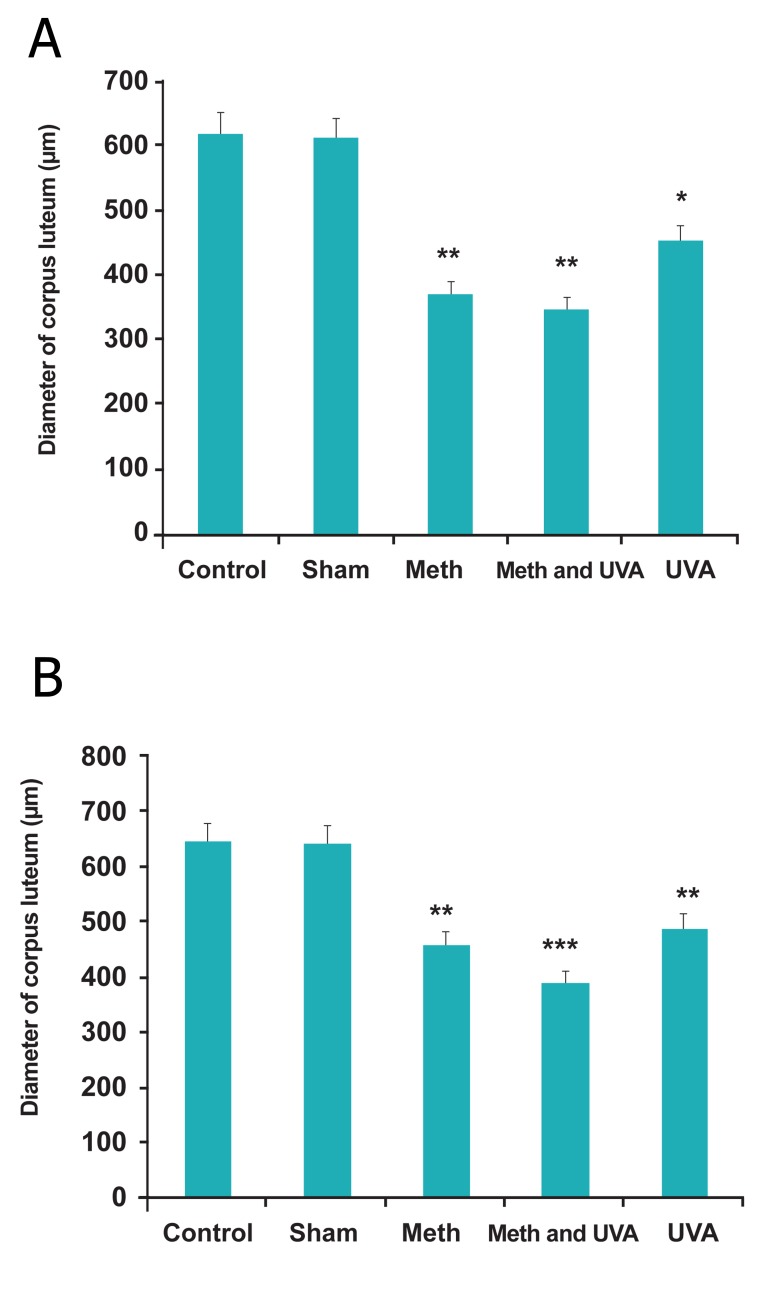
A. Comparision thickness of corpus luteum in long term methoxsalen injection with and without UVA (*p<0.5, **p<0.01). B. Comparision thickness of corpus luteum in short term methoxsalen injection with and without UVA (**p<0.01, ***p<0.001).

**Fig 6:**
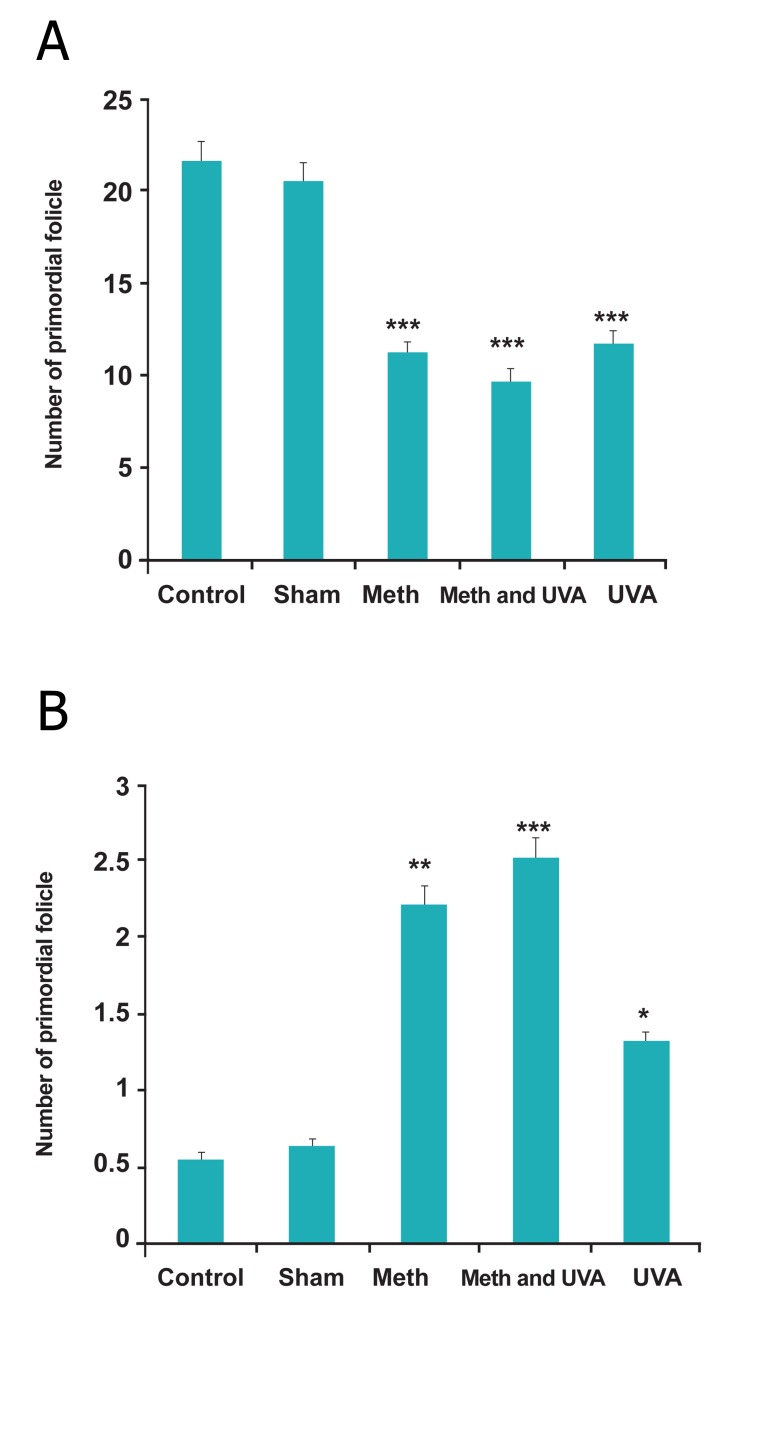
A. Comparision of primordial follicle in long term methoxslen injection with and without UVA (***p<0.001). B. Comparsion of primordial follicle in short term methoxsalen injection with andwithout UVA (*p<0.05, ***p<0.001).

**Fig 7:**
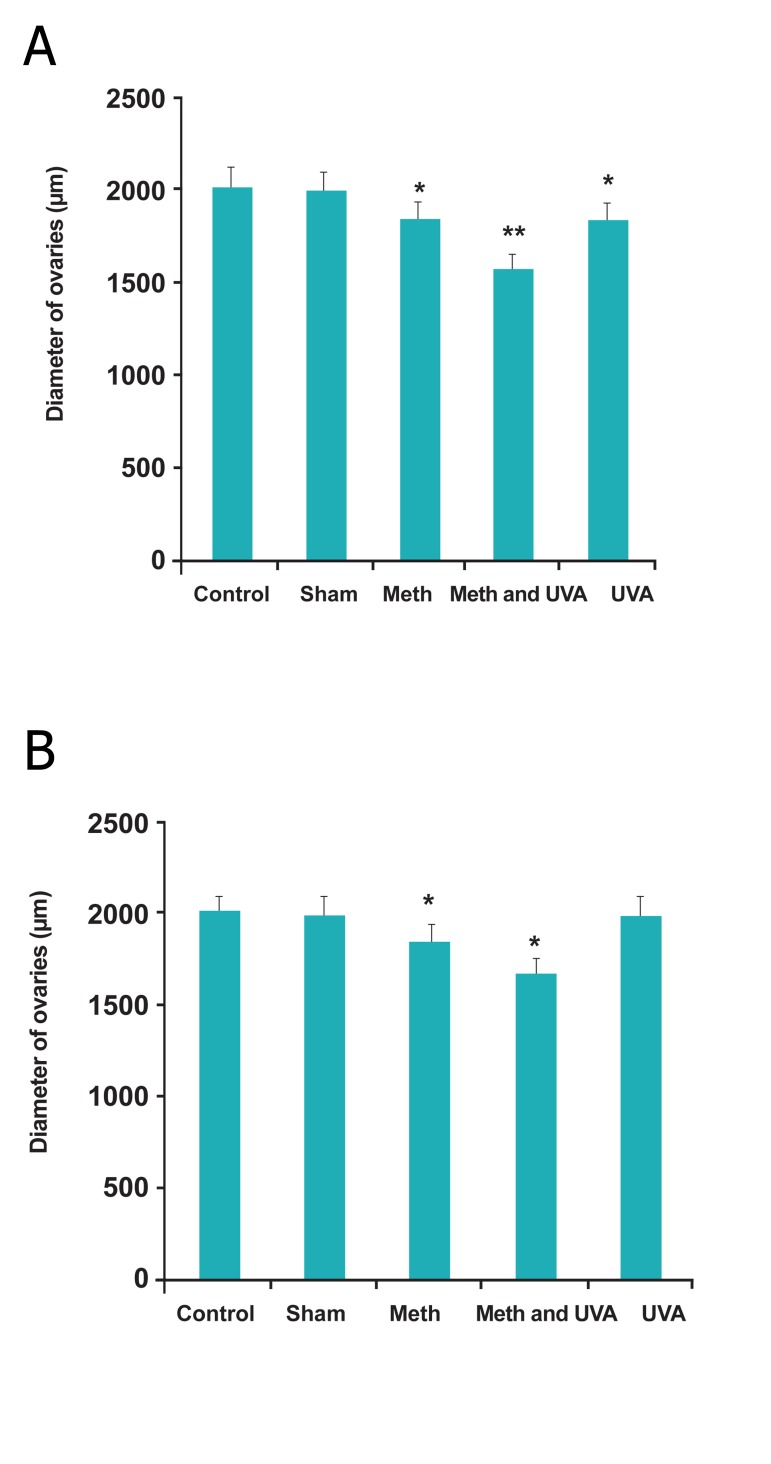
A. Comparision number of atretic follicle in long term methoxsalen injection with and without UVA (***p<0.001). B. Comparision number of atretic follicle in short term methoxsalen injection with and without UVA (*p<0.05, ***p<0.001).

**Fig 8:**
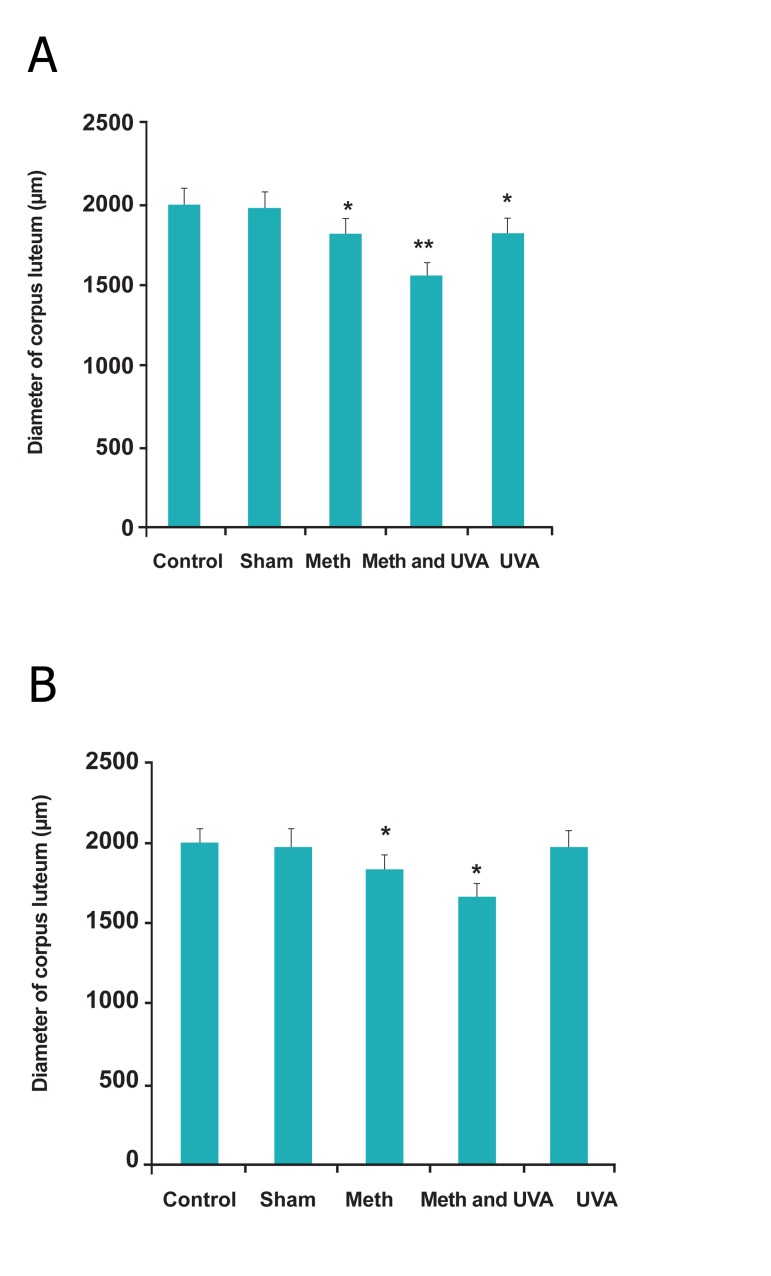
A. Comparision diameter of ovaries in long term methoxsalen injection with and without UVA (*p<0.05, **p<0.01). B. comparision diameter of ovaries in short term methoxsalen injection with and without UVA (*p<0.05).

As shown in figure 8A, B, diameter of ovaries reduced significantly in long and short term injections in all treatment groups, but the ovaries weight increased (p<0.05).

**Fig 9:**
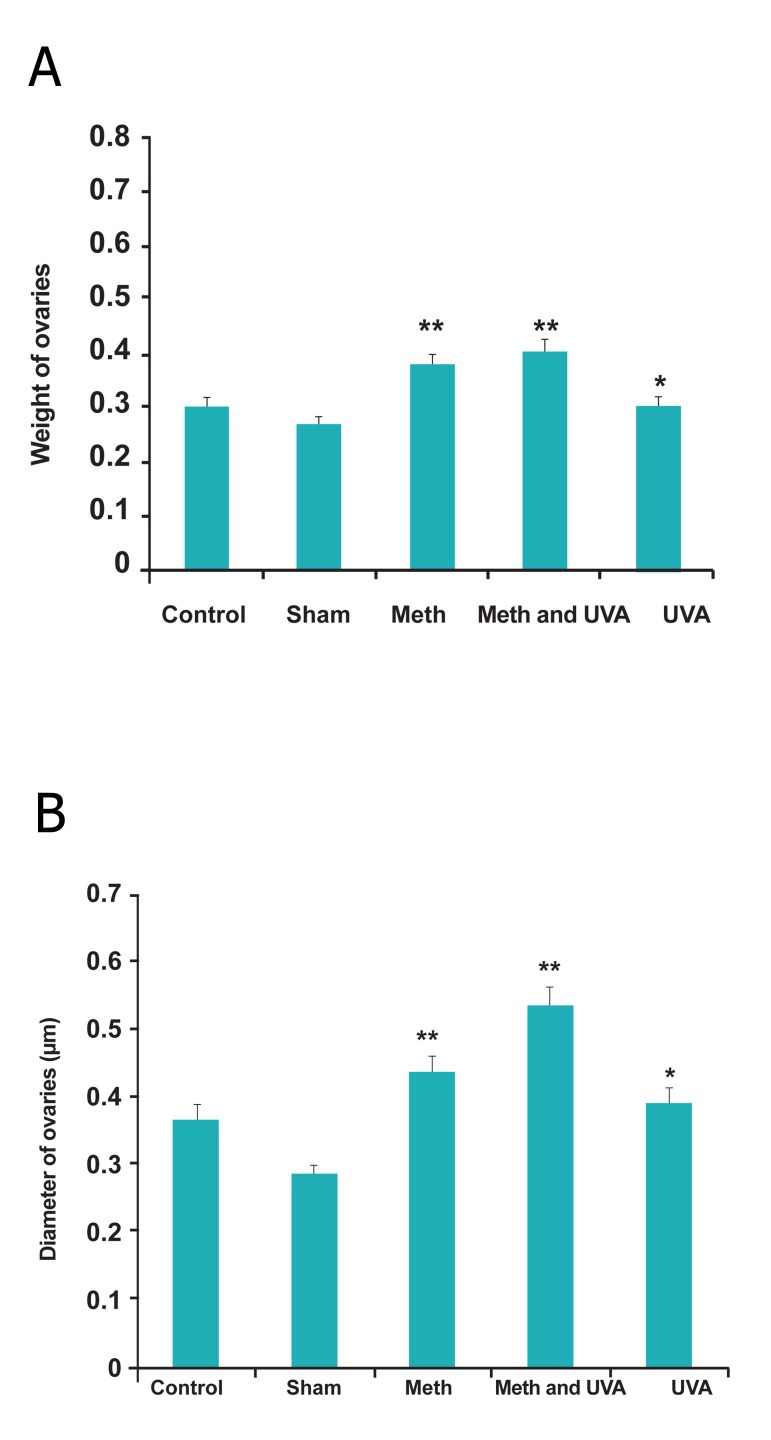
A. Comparision weight of ovaries in long term methoxsalen injection with and without UVA (* p<0.05, **p<0.01). B. Comparision weight of ovaries in short term methoxsalen injection with and without UVA (*p<0.05, **p<0.01).

The relative weight of ovaries increased in methoxsalen and methoxsalen plus UVA groups in long term injection, while it reduced in the same groups in short term, significantly (Fig 9A, B).

**Fig 10:**
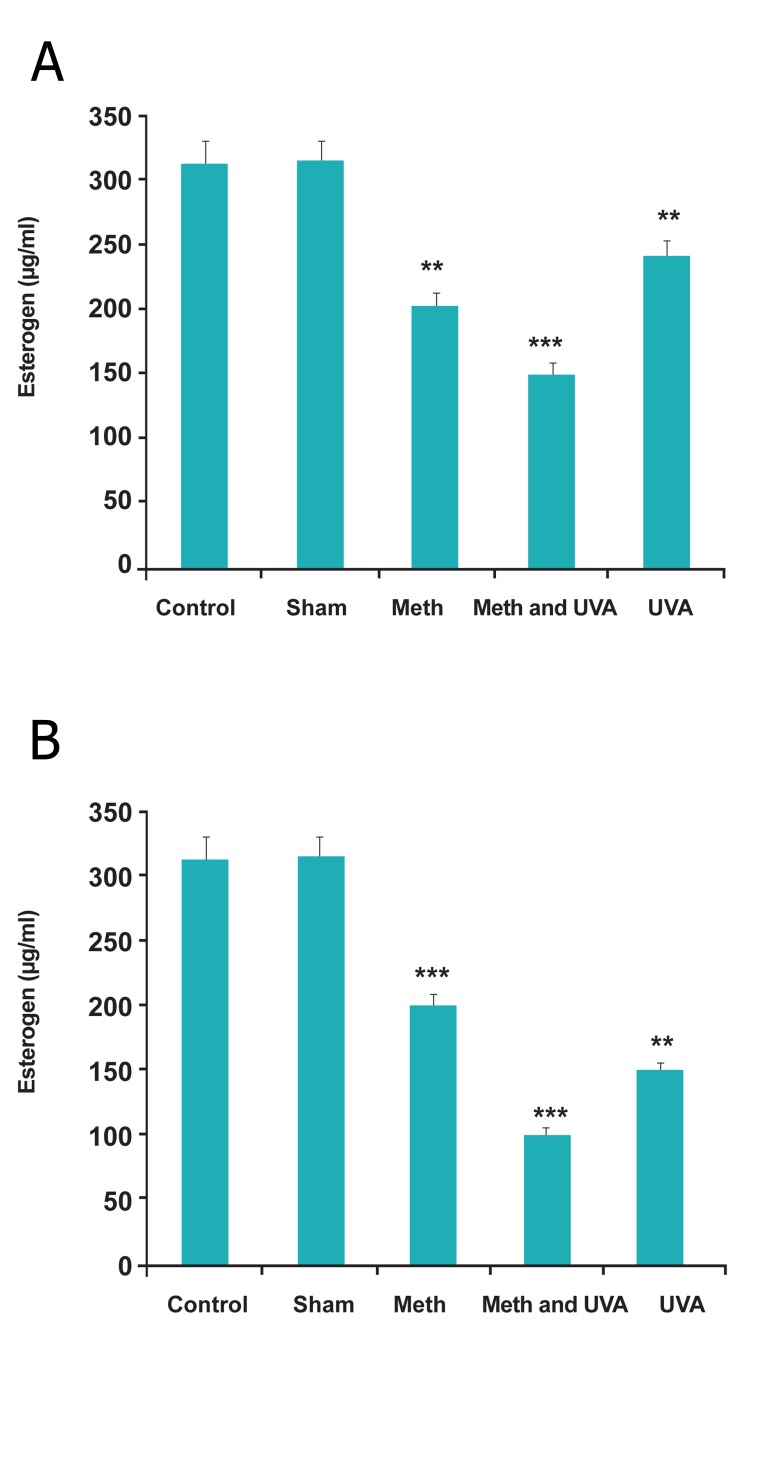
A. Comparision of esterogen in long term methoxsalen injection with and without UVA (**P<0.01, ***P<0.001). B. Comparision of esterogen in short term methoxsalen injection with and without UVA (**p<0.01, ***p<0.001).

Circulating estrogen levels in blood serum reduced in experimental groups as compared with the control group, significantly (Fig10A, B).

**Fig 11:**
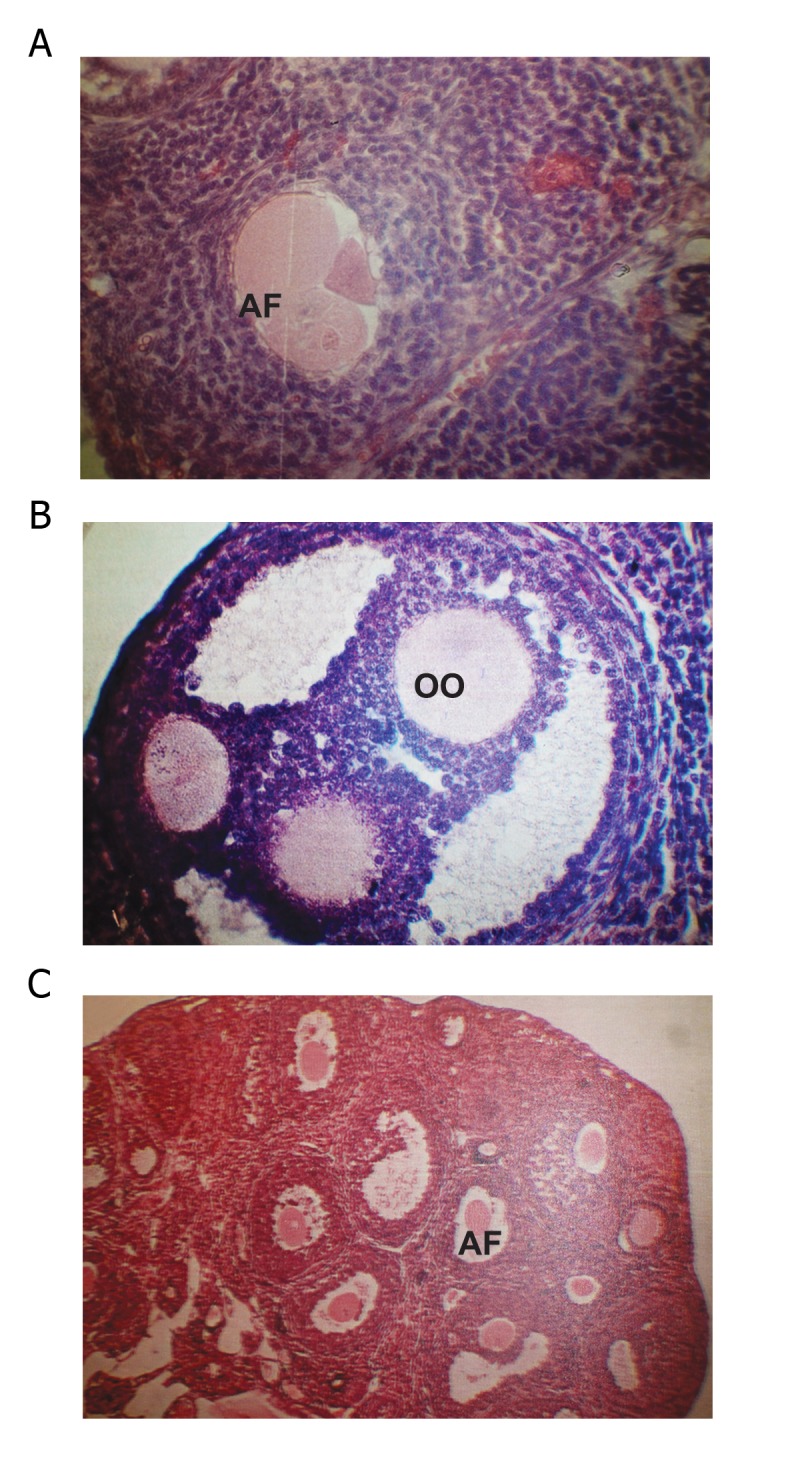
A. Photomicrograph of atretic follicle (AF) in long term methosxalen injection with UVA radiation. B. Photomicrograph of follicle content of three oocytes in long term methoxsalen with UVA injection. C. Photomicrograph part of ovary in long term methoxsalen injection and number of atretic follicle. ×250.

Some other cases of teratogencity such as follicles containing three oocytes and disorganization in corpus lutea cells were observed (Fig 11). Comparing the obtained results revealed that there were no significant differences between short and long term treatment among the experimental groups.

## Discussion

Methoxsalen is a photoactive compound which is used together with UVA in treatment of a number of epidermal proliferative disorders (vitiligo and psoriasis) (2). In this study, significant reduction was observed in the number and diameter of corpora lutea, number of implantation sites and Graafian follicle in response to the doses of methoxsalen, UVA and combination of them. Since the number of corpora lutea directly reflects the number of ovulated oocytes, these findings are consistent with reduction in number of ovulations. Uterus is considered as target organ for progesterone, so reduction in the number of corpora lutea causes progesterone reduction. The reduction in circulating estrogen levels by methoxsalen, UVA and methoxsalen plus UVA is also consistent with reduction in development or function of different follicles, like primary and primordial, which could impact on ovulation rate. Mono functional and bi functional adducts cause prevention of DNA synthesis and mitotic division, so replacement in germ cells will be delayed. UVA provided thymine-thymine cyclobutane dimer by cross-linking between side prymidine bases, preventing DNA synthesis in S phase. Methoxsalen plus UVA are directly functional by production of oxygen reactive species and oxidative lipids (20,21). UVA cause change in the activity of mitochondria by apoptosis. Recent studies have showed an increase in the diameter of theca by UVA treatment. Probably, after UVA treatment, theca stimulates cell growth and consequently increases in mitotic activity. In addition, UVA stimulates cell growth by an increase in cyclic adenosine monophosphate (cAMP) and 5'-guanylic acid (5'-GMP). In this study, serum level of estrogen in different experimental groups was reduced. Estrogen is secreted by granulosa cell layer, reduction of diameter of granulosa cell could directly effect on the estrogen levels of blood serum. There is a negative correlation between estrogen and induction of liver enzymes CYP1A1 and UGT1A6. It has been reported that estrogen is synthesized in ovarian granolusa cells by aromatase, which is a CYP450 dependent (15). Studies by Diawara et al. (12) demonstrated that psoralens inactivated mouse CYP1A1 *in vitro* using a mechanism based on the inactivation pathway. It is important to understand direct effect of the psoralens on the activity of this general class of enzymes. In addition, CYP1A1 and CYP1A2 are effective in hydroxylation of estrogen. In fact, CYP1A1 inducers lower circulating levels of estrogen in women, and have also been proposed as chemo preventive agents for estrogen dependent cancer. Other studies found that a single intraperitoneal (IP) injection of xanthotoxin to male Sprague Dawley rats induced liver CYP1A and CYP2B mRNA, proteins, and catalytic activities in a dose dependent manner (17). In contrast, Gwany (19) has reported that lack of induction of UGT2B1 mRNA and induction of CYP2B remains for further elucidation. Xanthotoxin are aryl hydrocarbon (Ah) receptor-dependent inducing agents. Induction of Ah- receptor-dependent indicates that CYP1A2 catalyzes the 2-hydroxylation of estrogen leading to the low circulating levels of estradiol, followed by infertility (13-18). The diameter of oocytes reduced in experimental groups as compared with the control, significantly. Our findings revealed that liver CYP1A2 enzymes caused a reduction in both diameters of granolusa and estrogen levels in blood serum.

An increase of atretic and disorganized follicles may be related to production of reactive oxygen by combination of UVA and methoxsalen which can destroy membrane lipids, and may affect the process of oogenesis and ovulation.

## Conclusion

These results suggest that UVA, methoxsalen and their combination cause health hazards and cell injury. The potential risk to humans must be more evaluated to ensure continued safe use of methoxsalen in photochemothrapy.
